# 31. CNS disease: is it infection, rheumatologic or an overlap of both?

**DOI:** 10.1093/rap/rky033.022

**Published:** 2018-09-20

**Authors:** Fatima Haji, Osama Alalwan

**Affiliations:** Rheumatology, Salmanya Medical Hospital, Manama, BAHRAIN

**Introduction:** Neuromyelitis optica (NMO, previously known as Devic disease) and neuromyelitis optica spectrum disorders (NMOSD) are inflammatory disorders of the central nervous system characterised by severe, immune-mediated demyelination and axonal damage predominantly targeting optic nerves and spinal cord. NMO is frequently associated with systemic autoimmune disorders. This includes systemic lupus erythematosus, antiphospholipid syndrome, and Sjögren's syndrome. Mixed connective tissue disease (MCTD) is an overlap syndrome combining features of systemic lupus erythematosus, systemic sclerosis, and polymyositis, together with the presence of antibodies to U1-RNP. Neurologic manifestations may occur up to 17% of patients with MCTD. The most common neurologic problems are trigeminal neuralgia, headache, and aseptic meningitis. We report a case of MCTD complicated with neuromyelitis optica (NMO) that was initially diagnosed two years back as CNS infection with listeria in the presence of +ve AQP4 antibody both in serum and CSF of the patient.

**Case description:** A 27 year old Bahraini female, who was previously healthy with no past medical illness, first time came to medical attention in 2000, when she was 10 years old. She presented at that time to the dermatology clinic with a history of popular eruption over both upper and lower lips for nine months. She was diagnosed as having verruca vulgaris which resolved later. In 2008, she presented to the rheumatology clinic when she was 18. She had history of inflammatory polyarthritis involving both wrists, the small joints of both hands and both knees. Her rheumatoid factor was positive with high inflammatory markers. There were no features of other connective tissue diseases. She was diagnosed as having seropositive rheumatoid arthritis and started on methotrexate and hydroxychloroquine together with low dose steroid. The patient chose to stop therapy as she was planning for pregnancy. She had her first baby one year later, after which she disappeared and follow-up was lost for around five years. In July 2014, she presented to our hospital at age of 24 with a history of epigastric abdomen pain and fever for three weeks. Her serum amylase was mildly raised (200 U/L normal range up to 118 U/L) and urinary amylase 3394 U/L (normal range less than 650 U/L). She was admitted under the surgical team with a diagnosis of acute abdomen pain most probably due to acute pancreatitis. After admission, ultrasound abdomen was unremarkable and showed normal pancreas and gallbladder. Her abdominal pain resolved after few days spontaneously. During her stay in hospital, she complained of non positional vertigo. Also she gave history of diplopia two days after admission. She was also was having fever spikes. She was assessed by the neurology team and was found to have bilateral positive cerebellar signs (action tremor and impaired finger-nose test with generalised increased deep tendon reflexes). Her plantar reflexes were downwards. Speech, muscle power and sensation were intact. CT brain was normal. MRI brain showed abnormal signal intensity and enhancement involving predominantly the medulla oblongata and to a lesser extent the cervicomedullary junction and upper cervical cord, with a smooth leptomeningeal enhancement around the brainstem and upper cervical cord. Lumbar puncture showed lymphocytosis and increase in protein. She was started on IV ampicillin and IV acyclovir. After a few days her neurologic function improved significantly and the fever disappeared. CSF for herpes simplex virus PCR and CSF oligoclonal bands were negative. Her serum listeria antibodies, type 1: O positive with a titre of 1: 100 + (normal less than 1: 50) and CSF for aquaporin 4 antibodies was positive with a titre of 1: 10 + (normal less than 1: 1) Her serum oligoclonal bands was negative with normal ACE level. Serum paraneoplastic CNS antibodies (Hu, Ri, Yo, Ma, Ma2, Tr, amphiphysin) were all negative. Serum aquaporin 4 antibodies were positive with a titre of 1: 50 + (normal less than 1: 10). The neurology team thought this was brainstem encephalitis; most likely infectious secondary to listeria monocytogenes. Acyclovir was stopped after 14 days and IV ampicillin was planned for a total of six weeks. During her stay in hospital, the patient was reviewed by our rheumatology team. At that time, the patient provided us with a history of polyarthralgia not associated with joint swelling. There was history of hair loss or Raynaud’s phenomena. There was no history of oral ulcers, skin rash or photosensitivity at that time. Her labs were: ANA positive: speckled pattern, titer 1: 640 ENA profile: strongly positive for U1RNP Ab positive for SSA Ab, dsDNA Ab positive 24.3 P-ANCA positive RF negative DCT negative. Based on this, she was re-diagnosed with MCTD. She was started on hydroxychloroquine. The MRI brain was repeated, which showed: sSignificant increase in the extent of the abnormal signal involving the brainstem with recent involvement of the pons and midbrain Improvement in the enhancement pattern. No enhancing parenchyma lesions could be seem in the current study. However, a minimally leptomeningeal enhancement was noted in the posterior fossa between the cerebellar fissures on sagittal cervical spine sequences. Multiple cervical and proximal dorsal cord non-enhancing lesions were noted. She was discharged later after completing IV ampicillin for six weeks and was kept on oral amoxicillin for another six weeks. She didn’t receive steroids during her admission. During her three month follow-up in the neurology clinic, the MRI brain again showed normal study with complete resolution of previous lesions. In September 2016, she was presented at age of 27 to A&E with a history of generalised body pain for one week. There was history of fever, abdomen pain, increased urine frequency and dysuria for five days. The patient also reported history of lower back pain. She denied chest pain or SOB. No history of new skin rash. On examination: vitals: temperature 39.3, Bp 115/68, HR 120, chest: clear, CVS: tachycardia, no murmur, abdomen: soft, lower abdomen tenderness, large red patch over left upper chest area, mild right hand interphalangeal joints swelling. Her investigations: WBC 6.59, Hb 11.2, Plt 239, urea 5, Cr 62, Na 136, K 4.4, HCO3 22, Ca 2.14, albumen 38, total bilirubin 12, ALP 74, ALT 238, GGT 58, serum amylase 42, ketone negative, urine analysis: WBC 50-99 RBC 2-5, leukocyte esterase 2+. She was admitted on that day under general medicine care with impression of UTI. She was started on IV ceftriaxone. Urine and blood cultures sent. On third day of admission, she was noticed to have shortness of breath. Chest ray showed bilateral lower infiltrates. The impression was pneumonia. Clarithromycin was added to ceftriaxone. On next day, her SOB worsened. The responsible Firm requested Rheumatology consultation. Further history by rheumatology team revealed that she was having urinary retention initially together with dysuria. There was also a history of acute lower back pain and upper back pain at the level of the scapula. There was history of weakness and numbness of both lower limbs. There was no eye symptoms or upper limb weakness. Upon examination, she was sick looking, very dyspnic on 10 L/min oxygen via face mask. She was in severe pain and unable to change position. Chest: bilateral crepitations CVS: tachycardia, abdomen: soft, generalised tenderness, distended bladder, positive bowel sounds. Neurology exam: cranial nerves: bilateral gaze evoked nystagmus. Motor: normal power upper limbs Both lower limbs weakness (power 3/5). Increased DTR in all limbs plantar upward bilaterally, decreased sensation in right lower limb only. No sensory level. Based upon above findings and previous history, the impression of our rheumatology team was: to R/O neuromyelitis optica (NMO) secondary to MCTD presenting as NMO vs transverse myelitis. Urgent neurology team review (bilateral optic neuritis confirmed by endoscopy) and brain MRI requested. MRI showed long segment hyperintensity involving cervical cord from lower border of C3 to C6 level and also from T5 to T6 as well at T7-8 level. Subtle hyperintensity along hypothalamus and third ventricle subependymal aspect. CTPA done showed. No evidence of PE, bilateral mosaic ground glass opacities. Lumbar puncture was done showed WBC 0, culture was sterile. Protein 662 mg/L, (normal level 200-400). An echocardiography was done with study but was limited due to dyspnea and limited position. Findings were D-shaped septum with thin rim of pericardial effusion, LVEF 59%, RVSP couldn’t be estimated. The patient was moved to ICU. Ceftriaxone changed to meropenem and clarithromycin continued. Diagnosis of NMO secondary to MCTD was confirmed and methylprednisolone pulse 1 gm OD IV for five days started and the patient had five sessions of plasmapheresis. Serial CXRs were showing worsening bilateral infiltrate. She was kept on furosemide 40 mg BD IV. The respiratory and ID teams were consulted, their impressions were pneumonia associated with ARDS, possibly secondary to pneumocytis pneumonia (PCP) vs acute interstitial pneumonia secondary to MCTD. Trimethoprim-sulfamethoxazole and caspofungin and voriconazole were added. Later, blood and urine cultures results were negative. Respiratory profile was positive for chlamydia IgM antibobdy. DTA for PCP was negative. ProBNP was normal. After five days she was started on oral prednisolone 1 mg/kg po while still receiving multiple antibiotics (meropenem, clarithromycin, trimethoprim-sulfamethoxazole, caspofungin/voriconazole). The patient improved in her breathing and lower lib weakness. A week later she was out of ICU and able to walk with assistance. First dose of rituximab 1 gm was given. Two weeks later she was able to walk with a walking frame. SOB resolved with resolution of x-ray findings. She was discharged with tapering dose of prednisolone and a second dose of rituximab after two weeks from first dose. She was also scheduled to continue physiotherapy as outpatient. Later on, she was started on mycophenolate mofetil 1 gm BD with one year follow up with a complete resolution of her CNS symptoms and brain/spine MRI abnormalities and no evidence of recurrence of NMO attacks.

**Discussion:** Neuromyelitis optica, also known as Devic syndrome, is a rare autoimmune demyelinating disease of the central nervous system associated with inflammation of the optic nerve and longitudinally inflammatory lesions of the spinal cord. Presenting symptoms includes vision loss, pain with eye movement, bladder or bowel incontinence, hemiparesis, and paresthesias. Neurologic manifestations may occur up to 17% of patients with MCTD. The most common neurologic problems are trigeminal neuralgia, headache, and aseptic meningitis. Reported cases of MCTD complicated with neuromyelitis optica (NMO) are rare. The evaluation of suspected NMO requires brain and spinal cord MRI, determination of aquaporin-4 (AQP4) IgG serum autoantibody status, and often cerebrospinal fluid analysis. The detection of AQP4 IgG antibodies is specific for confirming the diagnosis in appropriate clinical settings. For patients with acute or recurrent attacks of NMO, initial treatment with high-dose IV methylprdnisone (1 gram daily for three to five consecutive days) is recommend. For patients with severe symptoms, unresponsive to glucocorticoids, treatment with plasma exchange is suggested. Also long-term immunosuppression treatment with azathioprine, rituximab or mycophenolate mofetil is recommended for the prevention of attacks as soon as the diagnosis is made.

**Key Learning Points:** CNC disease diagnosis can be difficult sometimes. Infection can precede or be superimposed in patients with connective tissue disease and can make diagnosis difficult especially when deciding on treatment with antibiotics or immunosuppresive drugs, and sometimes both may be needed. Comprehensive history and examination, the evaluation of suspected NMOSD entails brain and spinal cord neuroimaging with MRI, determination of AQP4 antibody status, and often cerebrospinal fluid analysis is need to reach diagnosis promptly. Multidisciplinary team input to reach the proper diagnosis and plan the treatment is a must in these cases.

**Disclosure: F. Haji:** None. **O. Alalwan:** None.

